# Replication Study of ESCC Susceptibility Genetic Polymorphisms Locating in the *ADH1B-ADH1C-ADH7* Cluster Identified by GWAS

**DOI:** 10.1371/journal.pone.0094096

**Published:** 2014-04-10

**Authors:** Jiwen Wang, Jinyu Wei, Xiaoling Xu, Wenting Pan, Yunxia Ge, Changchun Zhou, Chao Liu, Jia Gao, Ming Yang, Weimin Mao

**Affiliations:** 1 Cancer Research Institute, Zhejiang Cancer Hospital, Hangzhou, Zhejiang Province, China; 2 Key Laboratory of Diagnosis and Treatment Technology on Thoracic Oncology, Zhejiang Province, China; 3 College of Life Science and Technology, Beijing University of Chemical Technology, Beijing, China; 4 Clinical Laboratory, Shandong Cancer Hospital, Shandong Academy of Medical Sciences, Jinan, Shandong Province, China; 5 Clinical Laboratory, Zhejiang Cancer Hospital, Hangzhou, Zhejiang Province, China; 6 Clinical Laboratory, Cancer Institute and Hospital, Chinese Academy of Medical Sciences and Peking Union Medical College, Beijing, China; 7 Thoracic Surgery Department, Zhejiang Cancer Hospital, Hangzhou, Zhejiang Province, China; MOE Key Laboratory of Environment and Health, School of Public Health, Tongji Medical College, Huazhong University of Science and Technology, China

## Abstract

China was one of the countries with highest esophageal squamous cell carcinoma (ESCC) incidence and mortality worldwide. Alcohol drinking has been identified as a major environmental risk-factor related to ESCC. The alcohol dehydrogenase (ADH) family are major enzymes involved in the alcohol-metabolizing pathways, including alcohol dehydrogenase 1B (ADH1B) and ADH1C. Interestingly, *ADH1B* and *ADH1C* genes locate tandemly with *ADH7* in a genomic segment as a gene cluster, and are all polymorphic. Several ESCC susceptibility single nucleotide polymorphisms (SNPs) of the *ADH1B-ADH1C-ADH7* cluster have been identified previously through a genome-wide association study (GWAS). In the study, we examined the association between five *ADH1B-ADH1C-ADH7* cluster SNPs (rs1042026, rs17033, rs1614972, rs1789903 and rs17028973) and risk of developing ESCC. Genotypes were determined in two independent case-control sets from two regions of China. Odds ratios (ORs) and 95% confidence intervals (CIs) were estimated by logistic regression. Our data demonstrated that these *ADH1B-ADH1C-ADH7* cluster SNPs confer susceptibility to ESCC in these two case-control sets, which were consistent to results of the previous GWAS.

## Introduction

China was one of the countries with highest incidence and mortality of esophageal squamous cell carcinoma (ESCC) worldwide [1]. Epidemiological studies show that consumption of tobacco and alcohol are major risk factors for ESCC [2], [3]. However, only a portion of individuals exposed to tobacco and alcohol develop ESCC, indicating the crucial role of host susceptibility factors in ESCC. Accumulated evidences suggested that single nucleotide polymorphisms (SNP) might explain individual differences of susceptibility to ESCC through the candidate gene approach or the genome-wide association study (GWAS) approach [4]–[17].

Alcohol drinking has been identified as a major environmental risk-factor related to ESCC [2], [3]. Ethanol is metabolized *in vivo* by alcohol dehydrogenase (ADH) family and aldehyde dehydrogenase (ALDH), which are all polymorphic in human beings [18]–[19]. The total activity of ADH is significantly higher in cancer tissue than in healthy mucosa [18]. The *ALDH* and *ADH* polymorphisms influence individual diversity in alcohol-oxidizing capability and drinking behavior [19]. Among the ADH family, the major enzymes involved in the alcohol-metabolizing pathways are alcohol dehydrogenase 1B (ADH1B) and ADH1C. ADH1B and ADH1C exist as several homo- and heterodimers of ADH1A subunits, exhibit high activity for ethanol oxidation and play an essential role in ethanol catabolism. ADH7 is also a member of the ADH family. Although less efficient in ethanol oxidation compared to ADH1B or ADH1C, ADH7 is the most active as a retinol dehydrogenase. Therefore, ADH7 may take part in the synthesis of retinoic acid, a hormone important for cellular differentiation. Interestingly, the aforementioned three genes locate tandemly in a genomic segment as a gene cluster. Wu et al. identified several new ESCC susceptible SNPs, including *ADH1B* rs1042026 and rs17033, *ADH1C* rs1614972 and rs1789903 as well as *ADH7* rs17028973 through a GWAS based on analyses of in 2031 ESCC cases and 2044 controls with independent validation in 8092 ESCC cases and 8620 controls [4]. Considering the importance of *ADH1B*-*ADH1C*-*ADH7* cluster in ESCC, we conducted this replication case-control study to validate the association between *ADH1B* rs1042026 and rs17033, *ADH1C* rs1614972 and rs1789903 as well as *ADH7* rs17028973 SNPs and ESCC risk.

## Materials and Methods

### Study subjects

This study consisted of two case-control sets: (a) Hangzhou set: 617 patients with ESCC from Cancer Research Institute, Zhejiang Cancer Hospital (Hangzhou, Zhejiang Province, China) and sex- and age-matched (±5 years) 537 controls. Patients were recruited between January 2012 and March 2013 at Zhejiang Cancer Hospital. Control subjects were individuals who underwent a physical examination in the same hospital during the same time period as the patients were collected. (b) Jinan study: 540 patients with ESCC from Shandong Cancer Hospital, Shandong Academy of Medical Sciences (Jinan, Shandong Province, China) and sex- and age-matched (±5 years) 550 controls. Patients were recruited between June 2009 and April 2012 at Shandong Cancer Hospital. Control subjects were randomly selected from a pool of 4500 individuals from a community cancer-screening program for early detection of cancer conducted in Jinan city during the same time period as the patients were collected. The diagnosis of all patients was histologically confirmed. Individuals who smoked one cigarette per day for over 1 year were considered as smokers. Subjects were considered as alcohol drinkers, if they drank at least once per week. All subjects were ethnic Han Chinese. At recruitment, the written informed consent was obtained from each subject and each participant was then interviewed to collect detailed information on demographic characteristics, such as sex and age, and related risk factors, such as cigarette smoking, and alcohol drinking. This study was approved by the Review Board of Zhejiang Cancer Hospital and the Review Board of Shandong Cancer Hospital.

### SNP selection and genotyping

A total of five *ADH1B-ADH1C-ADH7* cluster SNPs (rs1042026, rs17033, rs1614972, rs1789903 and rs17028973) were included in the current study. These SNPs are ones identified by a previous ESCC GWAS in Chinese Han population [4]. All *ADH1B*-*ADH1C*-*ADH7* cluster SNPs were analyzed by the MassArray system (Sequenom Inc., San Diego, California, USA). A 15% blind, random sample of study subjects was genotyped in duplicates and the reproducibility was 100%.

### Statistical analyses

Pearson's χ^2^ test was used to examine the differences in demographic variables and genotype distributions of five *ADH1B-ADH1C-ADH7* cluster SNPs between patients and controls. The associations between genotypes of these SNPs and ESCC risk were estimated by ORs and their 95% CIs computed by logistic regression models. All ORs were adjusted for age, sex, smoking or drinking status, where it was appropriate. We tested the null hypotheses of multiplicative gene-environment interaction and evaluated departures from multiplicative interaction models by including main effect variables and their product terms in the logistic regression model [4], [20]–[23]. A *P* value of less than 0.05 was used as the criterion of statistical significance, and all statistical tests were two-sided. All analyses were performed using Statistical Analysis System (version 9.0; SAS Institute) and SPSS 16.0 (SPSS Inc.).

## Results

In terms of median age and sex distribution, no statistically significant differences were found between ESCC patients and healthy controls for Hangzhou set and Jinan set (all *P*>0.05), indicating that the frequency matching was appropriate (Table 1). However, there are more smokers and alcohol drinkers were observed among ESCC cases compared with controls in Jinan case-control sets (both *P*<0.05). There are no data on smoking and drinking status of controls in Hangzhou case-control set.

**Table 1:** Distribution of selected characteristics among ESCC patients and healthy controls.

Variable	Hangzhou case-control set (Discovery set)			Jinan case-control set (Validation set)		
	Cases	Controls	*P* 1	Cases	Controls	*P* 1
	No. (%)	No. (%)		No. (%)	No. (%)	
	617	537		540	550	
Age (year)^2^			0.737			0.167
≤62(≤56)	311(50.4)	276(51.4)		271(50.2)	299(54.4)	
>62(>56)	306(49.6)	261(48.6)		269(49.8)	251(45.6)	
Sex			0.855			0.193
Male	532(86.2)	465(86.6)		428(79.3)	453(82.4)	
Female	85(13.8)	72(13.4)		112(20.7)	97(17.6)	
Smoking status			NC			<0.001
Yes	426(69.0)	NA		354(65.5)	285(51.8)	
No	191(31.0)	NA		186(34.4)	265(48.2)	
Drinking status			NC			0.001
Yes	413(66.9)	NA		300(55.6)	251(45.6)	
No	204(33.1)	NA		240(44.4)	299(54.4)	

Note: ESCC, esophageal squamous cell carcinoma; NA, not available; NC, not calculated.

1 Two-sided χ^2^ test.

2 Median ages of cases for Hangzhou case-control set and Jinan case-control set are 62 and 56 years.

Firstly, unconditional logistic regression analysis was utilized to detect associations between five *ADH1B-ADH1C-ADH7* cluster SNPs (rs1042026, rs17033, rs1614972, rs1789903 and rs17028973) and ESCC risk in Hangzhou discovery set (Table 2). All observed genotype frequencies in controls conform to Hardy–Weinberg equilibrium in Hangzhou set. Logistic regression analyses revealed that all five SNPs were significantly associated with ESCC risk (*ADH1B* rs1042026: allelic OR = 2.02, 95% CI = 1.66–2.47, *P*<0.001; *ADH1B* rs17033: allelic OR = 1.58, 95% CI = 1.18–2.11, *P* = 0.001; *ADH1C* rs1614972: allelic OR = 1.65, 95% CI = 1.36–2.00, *P*<0.001; *ADH1C* rs1789903: allelic OR = 1.77, 95% CI = 1.33–2.35, *P*<0.001; *ADH7* rs17028973: allelic OR = 1.61, 95% CI = 1.35–1.92, *P*<0.001) (Table 2). The *ADH1B* rs1042026 A allele, *ADH1B* rs17033 G allele, *ADH1C* rs1614972 C allele, *ADH1C* rs1789903 G allele,and *ADH7* rs17028973 T allele were showed to be risk alleles.

**Table 2:** Associations between candidate SNPs in the *ADH1B-ADH1C-ADH7* cluster and ESCC risk in Hangzhou case-control set (Discovery set).

#	Identity	Gene locus	Position^1^	Case	Common genotype (%)	Heterozygous genotype (%)	Rare genotype (%)	Allelic OR^2^ (95% CI)	*P*
1	rs1042026	*ADH1B*	100447489	ESCC	51.1	35.1	13.8	2.02(1.66–2.47)	<0.001
	G>A			Control	66.7	29.8	3.5		
2	rs17033	*ADH1B*	100447968	ESCC	76.3	23.7	0	1.58(1.18–2.11)	0.001
	A>G			Control	84.3	15.7	0		
3	rs1614972	*ADH1C*	100477178	ESCC	49.3	39.0	11.7	1.65(1.36–2.00)	<0.001
	T>C			Control	60.3	35.4	4.3		
4	rs1789903	*ADH1C*	100481064	ESCC	75.4	22.9	1.8	1.77(1.33–2.35)	<0.001
	C>G			Control	84.5	15.1	0.4		
5	rs17028973	*ADH7*	100541809	ESCC	38.4	42.5	19.1	1.61(1.35–1.92)	<0.001
	C>T			Control	48.9	43.1	8.0		

Note: SNP, single nucleotide polymorphism; ESCC, esophageal squamous cell carcinoma; OR, odds ratio; CI, confidence interval.

1 Position in NCBI build 36.

2 Data were calculated by logistic regression.

Associations between genotypes of five *ADH1B-ADH1C-ADH7* cluster SNPs and risk of ESCC were estimated in Hangzhou discovery set (Table 3). Individuals with the *ADH1B* rs1042026 AG or AA genotype had an OR of 1.54(95% CI = 1.19–1.98, *P = *0.001) or 5.40(95% CI = 3.19–9.11, *P<*0.001) for developing ESCC, respectively, compared with individuals with the GG genotype (Table 3). *ADH1B* rs17033 AG carriers showed a 1.67-fold increased ESCC risk compared with those carrying the rs920778 CC genotype in two validation sets (95%CI = 1.24–2.26, *P* = 0.001) (Table 3). A significantly increased ESCC risk associated with the *ADH1C* rs1614972 TC or CC genotype compared with the TT genotype was observed (OR = 1.35; 95% CI  = 1.06–1.73, *P* = 0.016; OR = 3.59; 95% CI  = 2.19–5.88, *P*<0.001). The presence of the *ADH1C* rs1614972 CG or GG genotype was also associated with an increased risk of ESCC (OR = 1.70; 95% CI = 1.26–2.30 or OR = 5.50; 95% CI = 1.21–25.0, respectively) compared with the absence of such a genotype. Moreover, the *ADH7* rs17028973 TT genotype were significantly associated with increased risk of ESCC (OR = 3.07, 95% CI = 2.07–4.54, *P*<0.001). However, there was no such statistically significant association between the *ADH7* rs17028973 CT genotype and ESCC risk (OR = 1.25, 95% CI = 0.98–1.61, *P* = 0.078).

**Table 3:** Genotype frequencies of the *ADH1B-ADH1C-ADH7* cluster SNPs among cases and controls and their association with ESCC risk.

Genotypes	Hangzhou case-control set				Jinan case-control set			
	Cases, No. (%)	Controls, No. (%)	OR^1^ (95% CI)	*P* 1	Cases, No. (%)	Controls, No. (%)	OR^1^ (95% CI)	*P* 1
*ADH1B* rs1042026	*n* = 615	*n* = 537			*n* = 540	*n* = 550		
GG	314(51.1)	358(66.7)	1.00 (Reference)		285(52.8)	366(66.5)	1.00 (Reference)	
AG	216(35.1)	160(29.8)	1.54(1.19–1.98)	0.001	184(34.1)	165(30.0)	1.47(1.12–1.91)	0.005
AA	85(13.8)	19(3.5)	5.40(3.19–9.11)	<0.001	71(13.1)	19(3.5)	4.53(2.65–7.72)	<0.001
								
*ADH1B* rs17033	*n* = 617	*n* = 536			*n* = 540	*n* = 550		
AA	471(76.3)	452(84.3)	1.00 (Reference)		417(77.2)	464(84.4)	1.00 (Reference)	
AG	146(23.7)	84(15.7)	1.67(1.24–2.26)	0.001	123(22.8)	86(15.6)	1.58(1.16–2.16)	0.004
								
*ADH1C* rs1614972	*n* = 617	*n* = 537			*n* = 540	*n* = 550		
TT	302(49.3)	324(60.3)	1.00 (Reference)		271(50.2)	332(60.4)	1.00 (Reference)	
TC	239(39.0)	190(35.4)	1.35(1.06–1.73)	0.016	207(38.3)	195(35.4)	1.30(1.01–1.68)	0.045
CC	76(11.7)	23(4.3)	3.59(2.19–5.88)	<0.001	62(11.5)	23(4.2)	3.15(1.88–5.26)	<0.001
								
*ADH1C* rs1789903	*n* = 617	*n* = 536			*n* = 540	*n* = 550		
CC	465(75.4)	453(84.5)	1.00 (Reference)		413(76.5)	464(84.4)	1.00 (Reference)	
CG	141(22.9)	81(15.1)	1.70(1.26–2.30)	0.001	117(21.7)	83(15.1)	1.58(1.15–2.16)	0.005
GG	11(1.8)	2(0.4)	5.50(1.21–25.0)	0.027	10(1.9)	3(0.5)	3.49(0.94–13.02)	0.062
								
*ADH7* rs17028973	*n* = 617	*n* = 536			*n* = 540	*n* = 550		
CC	237(38.4)	262(48.9)	1.00 (Reference)		212(39.3)	270(49.1)	1.00 (Reference)	
CT	262(42.5)	231(43.1)	1.25(0.98–1.61)	0.078	232(43.0)	236(42.9)	1.26(0.97–1.63)	0.082
TT	118(19.1)	43(8.0)	3.07(2.07–4.54)	<0.001	96(17.8)	44(8.0)	2.62(1.75–3.93)	<0.001

Note: SNP, single nucleotide polymorphism; ESCC, esophageal squamous cell carcinoma; OR, odds ratio; CI, confidence interval.

1 Data were calculated by logistic regression with adjustment for age, sex, smoking and drinking status, where it was appropriate.

The association of ESCC risk with five *ADH1B-ADH1C-ADH7* cluster SNPs was further validated in an independent case-control set. Genotyping results showed that all five SNP were significantly associated with ESCC risk in Jinan Chinese population (Table 3). Carriers of the *ADH1B* rs1042026 AG or AA genotype showed significantly and consistently increased risk to develop ESCC compared with GG carriers (OR = 1.47, 95% CI = 1.12–1.91, *P* = 0.005; OR = 4.53, 95% CI = 2.65–7.72, *P*<0.001) (Table 3). Similar results were found for *ADH1B* rs17033 AG genotype (OR = 1.58, 95% CI = 1.16–2.16, *P* = 0.004) (Table 3). The odds of having the *ADH1C* rs1614972 CC or TC genotype in patients was 1.30(95% CI = 1.01–1.68, *P* = 0.045) or 1.30(95% CI = 1.01–1.68, *P*<0.001) compared with the TT genotype (Table 3). Although *ADH1C* rs1789903 CG genotype was significantly associated with ESCC risk in the validation stage (OR = 1.58, 95% CI = 1.15–2.16, *P* = 0.005), rs1789903 GG genotype was not associated with ESCC risk (OR = 3.49, 95% CI = 0.94–13.02, *P* = 0.062). Additionally, *ADH7* rs17028973 TT carriers showed a 2.62-fold increased ESCC risk compared with those carrying the CC genotype in the validation set (95%CI = 1.75–3.93, *P*<0.001) (Table 3).

The ESCC risk associated with the *ADH1B-ADH1C-ADH7* cluster SNPs was further examined by stratifying for smoking status and alcohol drinking history due to the key role of these enzymes in metabolism of ethanol and other toxics in Jinan case-control set (Table 4 and 5). Interestingly, we found higher odds of those five polymorphisms for developing ESCC among smokers than those among non-smokers (Table 4 and 5). Similar results were also observed among alcohol drinkers except *ADH1B* rs17033 genetic variant (Table 4 and 5). However, no evident gene-smoking interaction or gene-drinking interaction exists in Jinan case-control set (Table 4 and 5). We also examined whether there are gene-environment interaction between five *ADH1B-ADH1C-ADH7* cluster genetic variants and age and sex, but the results were negative (data not shown).

**Table 4:** Risk of ESCC associated with the *ADH1B* rs1042026 and rs17033 SNPs by smoking status and drinking history in Jinan set.

Variable	*ADH1B* rs1042026				*P*_interaction_ 3	*ADH1B* rs17033				*P*_interaction_ 3
	GG^1^	AG+AA^1^	OR^2^ (95% CI)	*P*		AA^1^	GG^1^	OR^2^ (95% CI)	*P*	
Smoking status					0.566					0.963
No	104/177	82/88	1.58(1.06–2.35)	0.026		149/229	37/36	1.58(0.93–2.66)	0.089	
Yes	181/189	173/96	1.96(1.40–2.77)	<0.001		268/235	86/50	1.65(1.09–2.49)	0.017	
Alcohol drinking					0.545					0.412
No	126/193	114/106	1.61(1.13–2.30)	0.008		180/254	60/45	1.71(1.10–2.66)	0.017	
Yes	159/173	141/78	1.97(1.37–2.82)	<0.001		237/210	63/41	1.41(0.90–2.20)	0.134	

Note: SNP, single nucleotide polymorphism; ESCC, esophageal squamous cell carcinoma; OR, odds ratio; CI, confidence interval.

1 Number of patients with genotype/number of control subjects with genotype.

2 Data were calculated by logistic regression, adjusted for sex, age, smoking and drinking history, where it was appropriate.

3 *P* values for gene-environment interaction were calculated using the multiplicative interaction term in SPSS software.

**Table 5:** Risk of ESCC associated with the *ADH1C* rs1614972 and rs1789903 and *ADH7* rs17028973SNPs by smoking status and drinking history in Jinan set.

Variable	*ADH1C* rs1614972				*P*_interaction_ 3	*ADH1C* rs1789903				*P*_interaction_ 3	*ADH7* rs17028973				*P*_interaction_ 3
	TT^a^	TC+CC^1^	OR^2^ (95% CI)	*P*		CC^1^	CG+GG^1^	OR^2^ (95% CI)	*P*		CC^1^	CT+TT^1^	OR^2^ (95% CI)	*P*	
Smoking status					0.512					0.077					0.160
No	98/160	88/105	1.42(0.96–2.11)	0.081		149/218	37/47	1.28(0.78–2.11)	0.336		85/131	127/139	1.12(0.76–1.66)	0.556	
Yes	173/172	181/133	1.65(1.18–2.31)	0.003		264/246	90/39	2.10(1.36–3.25)	0.001		127/139	227/146	1.83(1.30–2.57)	0.001	
Alcohol drinking					0.892					0.528					0.647
No	119/179	121/120	1.47(1.04–2.08)	0.031		183/248	57/51	1.50(0.97–2.31)	0.065		98/146	142/153	1.37(0.97–1.94)	0.076	
Yes	152/153	148/98	1.55(1.09–2.20)	0.014		230/216	70/35	1.85(1.17–2.92)	0.008		114/124	186/127	1.63(1.15–2.32)	0.006	

Note: SNP, single nucleotide polymorphism; ESCC, esophageal squamous cell carcinoma; OR, odds ratio; CI, confidence interval.

1 Number of patients with genotype/number of control subjects with genotype.

2 Data were calculated by logistic regression, adjusted for sex, age, smoking and drinking history, where it was appropriate.

3 *P* values for gene-environment interaction were calculated using the multiplicative interaction term in SPSS software.

## Discussion

In the current study, we examined the association between five *ADH1B-ADH1C-ADH7* cluster SNPs (rs1042026, rs17033, rs1614972, rs1789903 and rs17028973) and risk of developing ESCC in a two-stage case-control design. In this replication study, we successfully validated results of a previous GWAS that these five SNPs confer susceptibility to ESCC [4]. However, no significant gene-smoking interaction or gene-drinking interaction between these *ADH1B-ADH1C-ADH7* polymorphisms and ESCC was observed in this Chinese Han population.

Several molecular epidemiological studies using a candidate gene approach indicated a set of SNPs associated with ESCC susceptibility, primarily variations that are related to alcohol metabolism [24]–[30]. As a powerful and successful tool to identify common disease alleles, GWAS could interrogate a large amount of tagging SNPs that serve as surrogates for untested common SNPs across the genome. In published GWAS of cancers of the upper aerodigestive tract, including ESCC in individuals of European [28], [30], Japanese [9] and Chinese [4], have shown that SNPs in the *ADH* genes contribute to susceptibility of ESCC. Our results in this study are consistent to these reports and highlight the importance of genetic variants of the *ADH* genes in ESCC development.

There might be several limitations in the current case-control study. First, because it was a hospital-based study and the cases were from the hospital, inherent selection bias may exist. Thus, it is important to validate these findings in a population-based prospective study from the same geographic regions. Second, the statistical power of our study may be limited by the sample size, especially for statistical analyses of gene-covariate interaction. Third, future studies will need to address the biological function of these polymorphisms in the genesis of ESCC.

In summary, our study elucidated that the *ADH1B-ADH1C-ADH7* cluster polymorphisms were associated with risk of ESCC in Chinese populations. Our data support the hypothesis that multiple ADH genes are involved in ESCC etiology and highlight the importance of genetic components in cancer development [31]–[41].
